# Rubeola and Its Treatment

**Published:** 1879-08

**Authors:** E. A. Cobleigh

**Affiliations:** Athens, Tennessee


ATLANTA
Medical and Surgical Journal.

VOL. XVII.] AUGUST—1879. [No. 5

### Original Communications.

### RUBEOLA AND ITS TREATMENT.

BY E. A. COBLEIGH, M.D., ATHENS, TENN.

During March of this year I noticed among my patients a tendency to roseolous rashes, with little or no systemic disturbance, and heard of many more cases which either fell to other physicians for treatment, or were not treated at all. Following this tendency to cutaneous disturbance, about the first week in April there came a negro family to our town from some point in Georgia where measles were prevalent, and soon after their arrival here most of the members of that family had the rubeolous disease. From this neucleus the affection spread among our colored population, attacking men, women and children indiscriminately, and remaining confined exclusively to the negroes, although many of them lived among the whites in other parts of our town, where they took down, and went through the disease to recovery, until the 7th of May, when the first white case occurred and was treated by myself, the patient being a drug clerk, about eighteen years of age. Nine days later the second white case, a male student in the University here, presented himself at my office with the premonitory symptoms well marked, and I sent him to his room to await the eruption, which appeared promptly. This patient was also an adult, about twenty years old. After this the infection seemed to gather force, and became locally epidemic in its character, a large proportion of the cases occurring among whites and adults of both sexes, especially young men and women attending the schools here. There were probably not less than a hundred and fifty, or perhaps two hundred cases, all told, a large proportion of which were so mild as to undergo only domestic treatment.

Thirty-four cases fell into my hands of every grade of society. In one, a boy of twelve years, there were severe and repeated convulsions. Many were complicated by intense gastric disturbance. All had the usual catarrhal symptoms, and a few had troublesome sequelæ. In two patients occular inflammation was early resultant in corneal ulceration, both being scrofulous and out of health. Mild pneumonitis also occurred in two cases, the lung being already involved in exudative inflammation when first I saw the patients. Fever ran high enough to produce active nocturnal delirium in three instances. Suppression of urine and complete aphonia, the latter persisting for some time after convalescence, happened once in a strumous child of eight years. Aural catarrh and tonsillar abscess each oocurred once, and in one case—a boy of hemorrhagic diathesis—profuse recurring epistaxis gave me considerable trouble.

A negro man had temporary partial hemiplegia, and another patient suffered from slight hemoptysis. Intestinal catarrh followed the rubeolous attack in a few infantile cases, and severe gastralgia troubled one of my adult patients for a fortnight after recovery from his measles, both induced by errors of diet. Cough and nausea were especially harrassing in nearly all the cases until relieved by treatment.

In only a small proportion of my cases was I called early enough. The distressing complications or sequelæ were what usually forced an application for professional aid, and were fully under headway when I first saw the patients. This accounts for the rather formidable list of unpleasant intercurrent affections and results given above.

At least such is my own opinion, founded on a not limited personal experience with rubeolous epidemics, as I have witnessed them in three different States. I believe that measles, properly handled from the *very outset*, is by no means the intractable affection (outside of armies and crowded public places) which it was formerly considered, and which it proved to be under the heating treatment and supermedication of "lang syne." And when I say *properly handled* I do not mean *medicated* particularly, for I esteem hygienic measures of a greater value than drugs. It is a matter of chagrin to the scientific practitioner to find this nineteenth century, with its boasted enlightenment and advanced civilization, so prolific of superstitions. Especially do the professions of medicine and theology meet these relics of the dark ages, among the laity with whom they deal, in matters pertaining to their several sciences. And our predecessors in the professions have been largely responsible for the primary widespread inculcation of these very ideas which, because false and injurious in foundation or results, we to-day, at a great disadvantage, have to contend against with the masses.

The question, "What has been done for this patient?" in cases of measles, is generally met by the stereotyped answer, "given tea." As a rule, further inquiry develops the fact that the time-honored decoction politely dubbed "meadow saffron," (more vulgarly known as "sheep tea") was the article used, and almost invariably either without benefit or with positive injury. Enlightened America sneers at the Arab, with his balm of a thousand virtues, *punk*—the excrement of the camel—or its rival, the "dung of the sacred cow," so much resorted to for every variety of ailments by the pious Mahommedan, and yet our sentimental propriety revolts not at the use of the filthy and utterly inert "sheep tea." I speak in this matter, of course, only as regards the people—their original ideas on these subjects having, however, been derived from professional teachings, the custom being by no means limited to the ignorant classes.

But do the people alone entertain these erroneous views of the treatment of measles by teas and heat? Not at all, unless my intercourse with physicians—and it has been somewhat extensive—is exceptional. While I feel assured that the great body of my professional brethren have repudiated the old pathology with its resultant theories of therapeutics, and no longer treat the labored, over-heated patient by more heat, more stimulation, more goading, there are those, and the number is not small, who either continue the practice wholly or else regard the exanthemata as deserving to be treated thus, while other essential fevers are combatted by cold and depressant agents.

One of my earliest medical experiences was with rubeola. I soon found that teas and heat generally failed to work as well in my hands as previous teaching had led me to expect, while cold and sedatives had seemed to answer better, as shown by results. Yet I had still the lurking dread of popular prejudice lest I should "drive the eruption in," and dared not use decided cooling measures against the fever.

Further observation, and gradually bolder experimentation, led me to regard rubeola in the same light as any other light febrile disease, to fear no retrocession of the rash, and to direct my medication and remedial measures accordingly. My success has been more than satisfactory. The cold water treatment of scarlatina and typhoid, forced attention and final adoption, against the bitterest of opposition. No less applicable is it to the disease under consideration. Rubeola is a fever, an essential fever, often of high degree and marked fatality. An eruptive element does not alter the pathological essence of this disease. The danger lies in the temperature, primarily; in the complication and sequelæ, secondarily, and these latter mainly depend upon the intensity of the former. By this, I do not mean that every case of measles involves high fever, or threatens life. Far from it. It is generally a trivial affection, and the less we meddle with such cases the better. But no small proportion of patients either suffer much during the attack in non-malignant cases, (and their sufferings are deserving of mitigation) or are really endangered. Such should be treated and treated well; and most of these very cases are made so by meddlesome interference at the commencement of a mild attack. If we only relieve suffering and ward off ulterior results of unpleasant character, we do well; if we save life, we do better. One or the other of these things is possible of accomplishment in a vast majority of cases. What is the course to be pursued in order to obtain such results?

In mild cases, uncomplicated by any distressing symptoms—that is, where the catarrh and nausea are almost wanting, or are transient in character, the fever not high, bowels only slightly out of order and little or no cough, maintain a state of masterly inactivity; let the treatment be negative instead of positive; *inhibit emphatically* ALL *hot drinks;* allow moderate exercise and mild diet if patients wish it, (but as a general thing nature admonishes them to do without either, by preference;) let plenty of God's pure, fresh air and sunshine into the sick room, (if they are confined to a room, and this is not desirable unless in severe cold weather, or the invalid so chooses,) and, above all, give just as much COLD *water* to drink as *thirst calls for* and the stomach tolerates. With this plan, ninety per cent. of cases will not need any further attention, nor will complications arise. The rash will usually come out promptly and kindly, but if it proves wanting or abortive, no harm results. Eruption is not essential to the disease, to comfort, or to rapid and complete recovery. Too much importance is attached by physicians and patient to bringing out the rash. Let nature alone and it will be all right. Fill the interior of an already overheated body with hot tea; more heat, more internal irritation; more intense engorgement of the deeper organs and their vessels; more fuel to a consuming flame; and you are *not* driving the intensity of action to the surface, but directly calling it therefrom on the principle of revulsion. If the eruption comes, *as it generally will in spite of you*, it is no fault of yours, but a simple triumph of nature's processes in the face of obstructive interference. Cool the furnace, contract the surcharged and dilated capillaries, relieve the tension of vessel and nerve, calm the excitement, restore equilibrium, soothe and relax the irritated cutaneous glands and their ducts, do this by cold drinks *ad libitum*, and the patient's suffering are reduced to nil, or at least to a minimum, and the rash, if coming at all, will appear promptly. Iced lemonade, ice cream, iced lime-water and milk, or the cold milk alone, these are freely ordered for my patients, as their different gastric conditions indicate. Nausea is relieved and intolerable thirst quenched, mild cases of fever subside, complications are avoided, delirium prevented, rest secured.

In more severe cases, in *addition* to the foregoing, for I always resort to the above mentioned measures, I order warm, cool, or cold sponging or baths, according to the intensity of heat, *repeated frequently enough to control the thermal manifestations* and keep them within bounds. I have, in a few instances, resorted to the cold pack without hesitation, though this, as well as the baths, is not often necessary in so mild an affection as rubeola proves to be in the vast majority of cases. There need be no fear of retrocession of the rash, there must be no half way measures, if any malignant tendencies are recognized in the disease. You can't "*drive*" the eruption in if you try, at any stage of rubeola, nor will internal complications *follow* its retrocession. We have got the cart before the horse in this matter. Let inflammatory lesion of any gravity occur in the interior of the body, whether occult or plainly manifest, and in goes your rash, to use a common expression. Natural revulsion has been active, just as artificial counter-irritation from teas often accomplishes the same result. The complication is *followed* by retrocession, *not* retrocession by a complication.

Further, if the rash fades suddenly, in whole or in part, as I have seen it do often, and no evidences of mischief going on in the system are discoverable, let your patient severely alone, he is in no danger, is all right. I will cite a few cases to show the harmlessness of non-appearance, or disappearance of the rash.

G. H., male, aet. 23 years, full habit and never ill before. Had been broken out with measles for about three days, after a prodromic period of several days' duration. Was feeling rather under the weather; slight catarrh, cough, uneasiness in chest and coryza, but kept at work.

until to-day. Yesterday labored rather hard for a sick man, and taxed his strength to the point of fatigue. At 10 A.M. noticed that the eruption had entirely disappeared, but kept at work till night, though slightly weary all of the time. Retired and rested well; was doing nicely till eight this morning, when some friends frightened him by saying that if he did not drive the eruption out again he might die. Began at once drinking copiously of ginger tea, "sheep tea," ground ivy tea, and other things, taking to bed and covering up—the day was quite warm—so as to sweat, in which he succeeded at first; but perspiration soon ceased, fever ran high, gastralgia and spasm came on, abdomen swelled, and at six P.M. he sent for me. Found him in terrible agony, and excessively irritable. Gave emetic of zinc at once, followed by mustard water. He vomited enormous quantities of liquid, (had eaten nothing after breakfast) and then I gave him ice to suck, cool sponging, threw off the bed clothes, and ordered a dose of cathartic pills. In a short time he was gently perspiring, free from pain, abdominal tenderness gone, and the patient slept. Next day he was up, feeling comfortable; no return of the rash, and "no more tea in his'n" hereafter. This was on May 16th of the present year. His coryza and slight cough continued several days, and then subsided without treatment, and left him as well as before his illness.

E. S., female, aet. 17, had first symptoms of rubeola well marked on May 24th; put herself at once under my care, and was ordered cold drinks ad. lib., as usual with me, mild aperients and light diet. Case progressed mildly and well; was not confined to bed at all; eruption never came out except on neck and back, and not much there, but was quite characteristic of measles, and ran through the usual course.

F. K., male, aet. eight years, colored; symptoms of measles for ten days, during which time his mother has been assiduously plying him with the celebrated sheep extract. At dark on April 24th had two convulsions, one following the other in a few minutes. During the night had five more. Next morning I was called to the case to find him in prolonged spasm. Put him at once in a tub of warm water, and poured cold water on his head. Came out of the convulsion in a short time, and I immediately gave bromide of potassium grs. x. Ordered his teas to be stopped, cool sponging, iced drinks freely, a laxative, and potassium bromide every four hours. No more convulsions, fever moderated, patient easy, eruption came out that night in small isolated patches, never extended much over the surface, and soon disappeared, to leave the boy all right.

N. B., female, white, aet. 21 years; under treatment from the first. Never had any eruption except on one cheek and on anterior surface of the left arm. No grave symptoms, very little illness except intense nausea the first day, which yielded to aqua calcis, and prompt recovery without complications or sequelæ.

I could multiply examples, but these are enough for my purpose. Let the rash run its own uninterrupted course in treatment. Fight the *fever* by rational and direct febrifuge agencies, than which none are better than cold water and large doses of quinine when needed, the latter only as an adjunct to the former. Again, I insist that the eruption *can't be forced in* unless internal complications are first set going. My treatment then resolves itself into cold internally *always*, warmth to the surface in some not markedly febrile cases, cool or cold applications to the periphery, in degree and duration proportionate to the head increment when needed, and regardless of the rash; calmatives in some instances, as the bromides, opiates, acetate of ammonia, etc., for wakefulness, cough and nervous symptoms; lime water or cerium oxalate for nausea and vomiting; and mild laxatives when indicated. Complications must be met by remedies such as would be used, on general principles, for the affections to be combatted, if occurring idiopathically.

Of the cases mentioned in the first part of this article, nearly all complications there spoken of arose in persons who had been first medicated with hot teas, hot rooms, hot beds, hot baths, starved and purged according to popular custom. On commencing a radically opposite plan, they improved as by magic from the start, and without exception expressed or manifested immediate relief from what had previously been torture. In most cases the eruption was extensive while using the cold to interior or exterior or both; and in a few instances it was excessive, showing beyond controversy—and one fact is worth a hundred unsubstantiated theories, however plausible or scientific—that cold does not repel the cutaneous manifestations of rubeola or any other exanthem where nothing else interferes.

If physicians who are afraid of cold in measles will only try it once, they will lose all fear of the method—will be happily disappointed at the prompt recovery of their patients; will find not only their sufferings mitigated, but actually shortened as well, though I do not claim abortive powers for the antipyretic plan of therapeutics; will meet fewer troublesome complications and almost no pathological after-conditions.

